# Development of Bacterial Spore Pouches as a Tool to Evaluate the Sterilization Efficiency—A Case Study with Microwave Sterilization Using *Clostridium sporogenes* and *Geobacillus stearothermophilus*

**DOI:** 10.3390/foods9101342

**Published:** 2020-09-23

**Authors:** Aswathi Soni, Jeremy Smith, Richard Archer, Amanda Gardner, Kris Tong, Gale Brightwell

**Affiliations:** 1 Food Assurance, AgResearch, Palmerston North 4410, New Zealand; Amanda.Gardner@agresearch.co.nz (A.G.); Gale.Brightwell@agresearch.co.nz (G.B.); 2 School of Food & Advanced Technology, Massey University, Palmerston North 4410, New Zealand; J.R.Smith@massey.ac.nz (J.S.); R.H.Archer@massey.ac.nz (R.A.); K.Tong@massey.ac.nz (K.T.); 3 New Zealand Food Safety Science Research Centre, Palmerston North 4410, New Zealand

**Keywords:** microwave, sterilization, *Geobacillus*, *Clostridium*, spores, inactivation, thermal resistance, Maillard reaction

## Abstract

In this study, novel spore pouches were developed using mashed potato as a food model inoculated with either *Geobacillus stearothermophilus* or *Clostridium sporogenes* spores. These spore pouches were used to evaluate the sterilization efficiency of Coaxially induced microwave pasteurization and sterilization (CiMPAS) as a case study. CiMPAS technology combines microwave energy (915 MHz) along with hot water immersion to sterilize food in polymeric packages. The spore pouches were placed at pre-determined specific locations, especially cold spots in each food tray before being processed using two regimes (R-121 and R-65), which consisted of 121 °C and 65 °C at 12 and 22 kW, respectively, followed by recovery and enumeration of the surviving spores. To identify cold spots or the location for inoculation, mashed potato was spiked with Maillard precursors and processed through CiMPAS, followed by measurement of lightness values (*L-values). Inactivation equivalent to of 1–2 Log CFU/g and >6 Log CFU/g for *Geobacillus stearothermophilus* and *Clostridium sporogenes* spores, respectively was obtained on the cold spots using R-121, which comprised of a total processing time of 64.2 min. Whereas, inactivation of <1 and 2–3 Log CFU/g for *G. stearothermophilus* and *C. sporogenes* spores, respectively on the cold spots was obtained using R-65 (total processing time of 68.3 min), whereas inactivation of 1–3 Log CFU/g of *C. sporogenes* spores was obtained on the sides of the tray. The results were reproducible across three processing replicates for each regime and inactivation at the specific locations were clearly distinguishable. The study indicated a strong potential to use spore pouches as a tool for validation studies of microwave-induced sterilization.

## 1. Introduction

Any novel sterilization technology brings along the necessity to develop methods to ensure that the coldest regions inside the food would receive enough treatment to achieve the inactivation of microorganisms (mesophiles and thermophiles) to ensure food safety. Coaxially induced microwave pasteurization and sterilization (CiMPAS) is an emerging thermal technology that combines hot water immersion and microwave energy (915 MHz) to achieve sterilization in a shorter time as compared to the conventional technologies [1,2]. Microwave sterilization technology, originally developed at Washington State University [3], has been validated and accepted by the Food and Drug Administration (FDA) as a thermal sterilization for producing pre-packaged, low-acid foods [2]. CiMPAS model consists of a hot water tank, a warm water tank a process vessel (with microwave outlets) and an electric cabinet. The operation can be divided into four major steps namely, preheat, hot water treatment, holding and cooling. CiMPAS technique can be controlled using various factors including the power of the microwave, time of exposure and the cooling mechanisms. Microwave sterilization uses less processing time as compared to conventional retorting and thereby the exposure of the nutrients in food to high temperatures is reduced to allow better nutrient retention [1,2,4]. However, a challenge with microwave sterilization is the non-uniform heating that could lead to the formation of cold spots in the processed products [5,6]. The presence of cold spots or regions that have been less thermally processed might result in incomplete bacterial inactivation. Hot or cold spots may also lead to uneven cooking, consequent undesirable sensory properties and nutrient losses.

Cold spots in foods processed using microwave-induced sterilization technology have been identified and reported using chemical markers [7,8] and temperature probes in previous studies [7,9]. Chemical markers, for example, products of Maillard reaction can serve as local time-temperature integrators. Maillard reaction involves a reducing sugar (e.g., ribose) that condenses with a compound possessing a free amino group (e.g., amino acid) to give a product (N-substituted glucosamine) that gets further arranged to form an Amadori rearrangement product (ARP) followed by an array of chemical reactions leading to the formation of compounds that impart the brown color [10]. One of these products is the chemical marker M-2 (4-hydroxy-5-methyl-3(2H)-furanone), which has been reported as an effective tool to monitor heating patterns of foods after microwave sterilization [10]. However, until now, the microbial inactivation on these colder spots have not been investigated or reported. Several microbial inactivation assays are required to ensure reproducibility and reliability [11,12]. A common method to test sterilization regimes is the use of spore strips [13,14] Several microbial inactivation assays are required to ensure reproducibility and reliability [11,12]. A common method to test sterilization regimes is the use of spore strips [13,14] that can be placed inside the sterilization chamber. Though this has been used an effective way to monitor sterilization, it only indicates whether the pre-determined spore numbers were either completely inactivated or not, hence either indicates presence or absence of spores, but the surviving spores cannot be enumerated. Also, they cannot be directly used for challenge testing in food for localized inoculation and recovery. Thermal processing efficiency may also be monitored by the conventional way of inoculating whole trays of food with specific strains of bacterial spores and subsequently measuring Log reductions. However, it is not possible to recover these spores from unique locations post treatment within the bulk food and hence the inactivation efficiency cannot be related to back to the spatial distribution of the cold spots.

To address this research gap, the current study investigated the use of spore pouches that were developed using food model inoculated with bacterial spores packed in microwavable Cryovac BNB1 pouch (15 mm^2^) (Cryovac, Hamilton, New Zealand). These pouches could be placed at specific target locations within the packaging trays filled with homogeneous food, followed by microwave sterilization to recover and enumerate the spores that survive treatment. For the formulation of spore pouches, two different typed strains of bacterial spores were used; *Geobacillus stearothermophilus* ATCC 12980 and *Clostridium sporogenes* spores NZRM 3052. The selection of two different spores was based on the significant difference in their thermal resistance as per the previously reported *D* values, which were also further confirmed in the current study. Decimal reduction time or *D*-value is defined as the time required at any specific temperature to achieve inactivation equivalent to 1 Log CFU/mL of a specific bacterial population [15]. *D* values at 121 °C for *C. sporogenes* have been reported as 0.5 and 0.6 min in phosphate buffer (pH 7.0) and carrot juice, respectively [16]. *G. stearothermophilus* spores have been reported to have D values of up to 5.4 min at 121 °C in yeast extract media [17]. The thermal resistance of these spores would also depend on their sporulation conditions and the medium in which the inactivation takes place. Hence, spores were inoculated in mashed potato in the current study instead of a diluent or buffer while being processed in CiMPAS to keep any specific effect on the thermal resistance in consideration while interpreting the results. The main objective was to test the possibility of using spore pouches to evaluate sterilization efficiency of CiMPAS using inactivation of spores at specific regions (cold spots) inside food packaging trays. The method used to identify cold spots for spore inoculation was by comparing the difference in browning using the Lightness (L) values, which indicate the formation of a chemical marker (M2) that is one of the products of Maillard browning. As a case study, the CiMPAS system was operated in a manner to amplify inconsistencies within and between trays.

## 2. Materials and Methods

### 2.1. Preparation of Spores

*G. stearothermophilus* ATCC 12980 spores were produced by a method previously described by Sadiq et al. [18] with a slight modification. Briefly, an overnight culture was grown in tryptic soy broth (TSB) at 60 °C for 24 h followed by spread plating 200 μL of the overnight culture onto the sporulation agar plates. The sporulation agar plates (final pH 7.0) comprised of nutrient agar (NA; Difco) (13 g/L), MgSO4·7H_2_O, (0.51 g/L), KCl, (0.97 g/L), CaCl_2_·2H_2_O, (0.2 g/L); MnSO_4_·H_2_O, (0.003 g/L), FeSO4·7H_2_O, (0.55 mg/L) and additional agar (1.5 g/L). The inoculated plates were incubated for 14 days at 60 °C followed by harvesting using cold sterile water (3 mL) by scraping the entire growth surface using sterile L-shaped disposable plastic spreaders. The spores were harvested by centrifugation (8000× *g*, 10 min, 4 °C) and washed three times with autoclaved pre-cooled distilled water. The purified spore stock suspended in distilled water was then stored at 4 °C for up to a maximum of 7 days until used.

*C. sporogenes* NZRM 3052 spores were cultured and in the same way [18] with a few modifications. An overnight culture was grown in Fluid Thioglycolate (FTG) media (Fort Richards, Auckland New Zealand) at 35 °C for 24 h in an anaerobic chamber followed by spread plating 200 μL on to tryptic sheep blood agar (SBA) plates (Fort Richards, New Zealand). The plates were incubated in an inverted position at 37 °C for 7 days in anaerobic chambers with anaerobic environment generator packs (BD GasPak™ EZ pouch systems, Fort Richards, Auckland New Zealand) and an indicator strip (BBL™ GasPak™ Anaerobic Indicator Strip, Dry, Fort Richards, New Zealand). The colonies on the surface of the agar were then scraped using the L-shaped spreader with cold sterile water (3 mL) to remove the sticky portions. The slurry was then washed three times by centrifugation (8000× *g*, 10 min, 4 °C) using distilled water. The spore suspension was stored at 4 °C in an anaerobic chamber until used.

### 2.2. Product/Food Model Formulation

Mashed potato (food model) was prepared as previously reported by Soni et al. [8]. In short, to prepare 1000 g of mashed potato (food model), agar (Fort Richards, Auckland, New Zealand) (5 g) was added to boiling water (830 g) and mixed using a cake mixer at medium speed for 2 min followed by addition of potato flakes (150 g) while mixing continuously to avoid lumps. The mix was cooled to 60 °C, followed by addition of D-ribose (10 g) and lysine (5 g) and mixed for another 2 min. Ribose and lysine (1 and 0.5%, respectively) have been reported to show formation of brown color with increasing time in the presence of heat, which can be measured by colorimetry [8,19]. This final composition of food model was left to cool in the room temperature for 10 min and then filled into packaging trays (174 × 103 × 35 mm) to reach a total weight of 250 g, while excluding the weight of the tray (20 g). Trays were then placed in microwavable Cryovac BNB1 pouches and sealed (23 MPa, 2 s) in a Multivac C200 vacuum sealer and used for CiMPAS processing.

### 2.3. CiMPAS Processing

**Figure 1:**
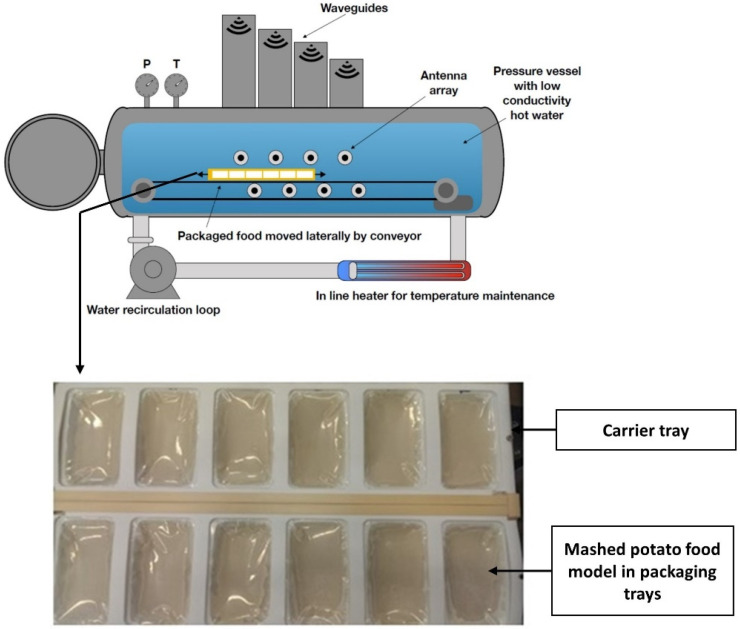
Schematic representation showing the mashed potato food model packed in the packaging trays and arranged in carrier tray while kept immersed in hot/warm water inside the pressure vessel in Coaxially induced microwave pasteurization and sterilization (CiMPAS).

CiMPAS system (Coaxially induced microwave-pasteurization and -sterilization) as previously described by Soni et al. [8] was manufactured by Meyer Burger Germany GmbH (Hohenstein-Ernstthal, Germany) and the industrial microwave parts were manufactured by MUEGGE GmbH (Reichelsheim, Germany). CiMPAS equipment used in the current study consists of a hot water tank, a warm water tank a process vessel (with microwave outlets) and an electric cabinet. The operation can be divided into four major steps namely, preheat, hot water treatment, holding and cooling. The sealed trays with mashed potato were placed in the CiMPAS carrier tray made up of polyether ether ketone (660 × 560 × 45 mm) which was then placed in the processing vessel (Figure 1) (filled with warm water with a conductivity of 8.4 uS/cm) for processing. A schematic representation of mashed potato food model in packaging trays placed in the carrier tray in the processing vessel is shown in Figure 1.

For CiMPAS processing, two processing regimes R-121 and R-65 were chosen to determine if spore pouches were able to indicate the difference in potential inactivation when processed through two different temperatures, microwave power and, hence processing times. CiMPAS regimes R-121 and R-65 used hot water at 121 °C and 65 °C to simulate sterilization and pasteurization, respectively. The carrier tray used here consists of 12 slots for packaging trays as shown in Figure 1. The detailed steps in the processing regime R-121 are explained in Table 1. For R121, following the preheating step, hot water (121 °C) was flushed into the vessel, microwave power was switched on at 12 kW, and the carrier tray was moved back and forth through the antennae as seen in Figure 1 for 250 s (Table 1) and the total processing time was 64.2 min.

The detailed steps in the processing regime R-65 is explained in Table 2. For R-65 with hot water at 65 °C was flushed into the vessel, microwave power was switched on at 22 kW and the carrier tray was moved back and forth for 500 s (Table 2) and the total processing time was 68.3 min.

CiMPAS regimes namely R-121 and R-65 could each only accommodate one carrier tray consisting of 12 packaging trays at a time (Figure 1). For both the regimes, as the final step cooling water (30 °C) was flushed into the vessel to cool the product. Processed packaging trays were removed from the carrier tray and placed into the chiller (4 °C) overnight before analysis. Samples were collected from three processing runs conducted on three different days separately for colorimetry and challenge testing. Controls were exposed to similar storage conditions except CiMPAS processing. Each processing run consisted of 12 samples and one control. The composition of mashed potato was not different in control; however, controls were not processed through CiMPAS and hence were untreated but were maintained at similar storage conditions along with samples for a direct comparison. The use of 12 trays for each processing run was entirely due to the machine set up where one carrier tray (Figure 1) consists of 12 slots, and hence to understand the spatial distribution of the processing effect, all the 12 slots were utilized.

### 2.4. Identification of Cold Spots for Inoculation by Colorimetric Analysis and High-Pressure Liquid Chromatography (HPLC) Analysis of Chemical Marker 4-hydroxy-5-methyl-3(2H)-furanone (M2)

After processing using CiMPAS, each tray was divided into nine different locations on the surface as previously described [8]. The lightness (*L values) were recorded using a Minolta CR20 colorimeter (Minolta Camera Co., Osaka, Japan) and using *Lab (CIELAB) space as previously reported [19,20]. The coldest spot on each tray was identified as the location with the significantly highest *L* values (*p* < 0.05) as the *L*-values reduce significantly as an effect of the increase in time when subjected to thermal treatment [8]. The increase in the brown color formation and hence the decrease in *L values has been previously validated using a kinetic study using oil bath set up at 121 °C [8]. To further verify the concentration of the chemical marker M2 at the cold spot, high-pressure liquid chromatography was used as previously described [9]. Mashed potato (food model) samples (1 g) was scooped out from the apparent cold and hot spots and were carefully ground using a mortar and pestle with an extraction buffer (10 mM sulphuric acid and 5 mM citric acid). The extracts were collected and stored overnight in a freezer (−18 °C), then thawed at room temperature before being centrifuged for 10 min at 10,897× *g*. The supernatants were collected and centrifuged again twice more to remove any debris, followed by filtration using a PTFE syringe filter (0.2 μm pore size) before being analyzed by HPLC. An Agilent 1100 HPLC system (Agilent Technology, Santa Clara, CA, USA) with diode array detector and an acid-fast analysis column (Bio-Rad Laboratories, Hercules, CA, USA) was used with a mobile phase flow rate of 1 mL/min. Absorbance was determined at 285 nm and a calibration curve was developed using the commercially available standard of M2 (Sigma, Castle Hill, NSW, Australia) with a concentration range of 0.0–1.4 mg/mL to interpolate the unknown concentrations of M2 in mashed potato (food model) extracts.

### 2.5. Estimation of Thermal Resistance at 121 °C (D Values) of C. sporogenes and G. stearothermophilus Spores

#### 2.5.1. Estimation of Decimal Reduction Time for *C. sporogenes* and *G. stearothermophilus* Spores in Milli-Q Water

An aliquot (50 μL) of the spore suspension in Milli-Q water was added to glass capillaries (diameter of 1.8 mm, length 70 mm), which were heat-sealed and immersed in Digital High-Temperature Oil Bath (Interlab, Wellington, New Zealand) pre-set at 121 °C as per the method described by Soni et al. [8]. The capillary tubes were removed from the oil bath at regular interval points (0, 2, 4, 6, 8 and 10 min) and immediately transferred to an ice slurry to stop the thermal inactivation. The tubes were washed once with a 90% ethanol solution and then twice with autoclaved distilled water before breaking the capillary tubes and transferring their contents into 0.1% peptone solution (*w*/*v*) for serial dilution. The number of spores present was determined by serial diluting the sample in 0.1% peptone and plating it onto sheep blood agar plates in triplicates. The trial was carried out in three experimental and three technical replicates. For *C. sporogenes* spores, capillary tubes were removed at a time interval of 1 min starting from 0 to 6 min whereas, for *G. stearothermophilus* spores, the time points were at an interval of 2 min ranging from 0 to 10 min based on the difference in thermal resistance. The inoculated plates were incubated and enumerated as described in Section 2.1.

#### 2.5.2. Estimation of Decimal Reduction Time for *C. sporogenes* and *G. stearothermophilus* Spores in Mashed Potato

*C. sporogenes* and *G. stearothermophilus* spores were separately inoculated in mashed potato to achieve a final inoculum of ~10^7^ and 10^6^ CFU/g respectively. A digital high-temperature oil bath (Interlab, Wellington, New Zealand) was set at 121 °C. The inoculated mashed potato (10 g) was filled in oil bath capsules that were previously reported [8] and sealed followed by immersion for incubation in the oil bath (121 °C) while making sure that there was no dripping or leakage from the capsules. The come-up time for mashed potato was 4 min and capsules were then removed at specific time intervals and transferred into ice-slurry for cooling down. For *C. sporogenes* spores, the capsules were removed at a time interval of 2 min starting from 0 to 10 min whereas, for *G. stearothermophilus* spores, the time points were at an interval of 4 min ranging from 0 to 16 min based on the difference in thermal resistance. Three experimental replicates were conducted under the same setup once cooled down, the surviving *G. stearothermophilus* spores in each sample were recovered by serially diluting and plating onto tryptic soy agar (TSA) plates. The plates were incubated in an inverted position at 60 °C for 48 h before the colonies on each plate were enumerated. *C. sporogenes* spores were serially diluted and plated onto tryptic SBA plates for enumeration. The plates were incubated in an inverted position at 37 °C for 48 h in anaerobic chambers (Section 2.1) followed by enumeration.

### 2.6. Inoculation of Bacterial Spores in the Food Model

For the challenge testing, *G. stearothermophilus* spores were inoculated using two methods: spot inoculation (using pouches) and whole tray inoculation and *C. sporogenes* spores were tested only using spot inoculation using spore pouches.

#### 2.6.1. Spot Inoculation of *G. stearothermophilus* Spores in Pouches

**Figure 2:**
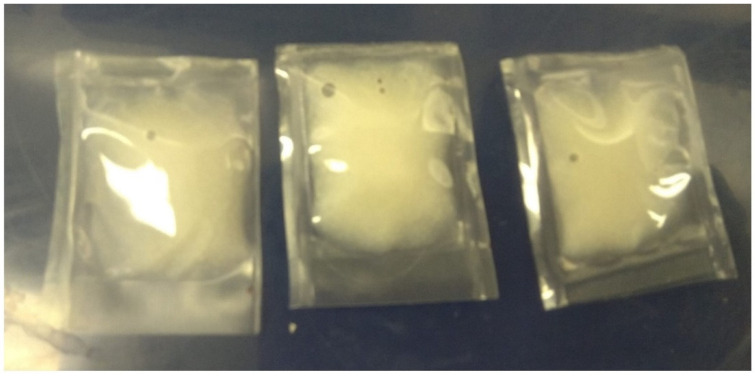
Microwavable pouch (Cryovac BNB1, Barrier Bag–70 Micron, 150 mm × 200 mm packages) containing mashed potato (1 g) inoculated with *G. stearothermophilus* spores followed by sealing on the four ends using HI Impulse Handsealer (Makmar, Auckland, New Zealand) with a maximum sealing thickness of 0.15 mm and a seal width of 2 mm and a sealing time of 2 s.

The original microwavable Cryovac BNB1 pouch (70 Micron, 150 mm × 200 mm) was cut into square-shaped pieces (15 mm^2^) and heat-sealed (HI Impulse Handsealer, Makmar, Auckland, New Zealand) with a maximum sealing thickness of 0.15 mm and a seal width of 2 mm with a sealing time of 2 s. Three sides of the pouch were sealed followed by placing 1 g of mashed potato (food model) inoculated with *G. stearothermophilus* spores (10^6^ CFU/g) followed by sealing the fourth side (Figure 2).

These spore pouches were then placed in the desired locations inside the packaging tray already filled with mashed potato food model for challenge testing. The target locations for the inoculation were the cold spot on each tray that were pre-determined in Section 2.4. For each tray, the cold spot was separately identified as the spot with significantly higher *L values (*p* < 0.05) after three CiMPAS processing runs.

#### 2.6.2. Inoculation of *G. stearothermophilus* Spores in the Whole Tray of Mashed Potato (Food Model)

For the homogeneous inoculation, 250 g of mashed potato (food model) was inoculated with 1.5 mL of *G. stearothermophilus* spores (10^6^ CFU/g), mixed for 2 s using a stomacher machine (Seward, Inc., London, England) followed by evenly spreading on to processing trays followed by packing and sealing as described in Section 2.2. For both pouch and whole tray inoculation using *G. stearothermophilus* spores, three processing replicates, were included, and three technical replicates used while plating each dilution. The processing replicates refer to three individual CiMPAS processing runs for the same regime that was conducted on separate days. Technical replicates refer to the use of three plates for spread plating and recovery for each dilution in each sample being analysed. The same replicate scheme was employed for the three studies.

#### 2.6.3. Spot Inoculation of *C. sporogenes* Spores

For the spot inoculation, *C. sporogenes* spores were inoculated in mashed potato and packed in pouches to achieve an inoculum level of 10^7^ CFU/g as described in Section 2.6.1. The spore pouches inoculated with *C. sporogenes* spores were used for spot inoculation in three separate trials. Firstly, R-65 was used to identify the cold spot (highest *L value), medium heated spot (intermediate *L value) and hot spot (lowest *L value) on each of the 12 trays. In the next trial, one spore pouch was placed each at the hot, cold and medium heated spot inside the packaging tray filled with mashed potato (food model) followed by CiMPAS processing by R-121 (Table 1), which took of a total time of 64.2 min. For the second study, an inoculated pouch was placed on the coldest location (on each of the 12 packaging trays) pre-determined by *L*-values (after R-65 in a previous run) followed by CiMPAS processing by R-65 (Table 2), which took of a total time of 68.3 min. For the third study, four pouches inoculated with *C. sporogenes* spores were placed vertically on the four sides/walls of each tray, followed by filling with mashed potato (food model) to obtain a total weight of 250 g before being sealed as described in Section 2.2 and processed via R-65. For each of these three studies, three processing replicates, were included, and three technical replicates used while plating each dilution as described in 2.6.2 and the same replicate scheme was employed for the three studies.

Whole tray inoculation trials were not conducted with *C. sporogenes* spores as ~7 Log reduction in spore pouches were achieved using the preliminary trials and with the whole tray studies, the inactivation would go below detection limit of 1 CFU/mL.

In summary, for challenge testing, *G. stearothermophilus* spores were subjected to both pouch and whole tray inoculation and were processed through R-121. *C. sporogenes* spores were inoculated only in spore pouches and subjected to microwave sterilization via R-65 and R-121.

### 2.7. Enumeration of Surviving Spores

To enumerate the surviving *G. stearothermophilus* spores in the pouches, each pouch was washed thrice in autoclaved distilled water to remove any residual food sticking on the surface followed by cutting on an edge using a sterile knife to empty the contents into a universal tube with peptone solution (0.1%, 9 mL) (Fort Richards, Auckland, New Zealand). To enumerate the spores surviving in mashed potato (food model) trays that were homogenously inoculated, the contents were first inverted into a sterile stomacher bag (1000 mL) and a sterile L-spreader was used to scrape off any leftover mashed potato (food model) for complete recovery. In both cases, the samples were mixed using a stomacher machine (Seward, Inc., London, England) for 2 s followed by serially diluting (10^−0^ to 10^−4^) and plating onto tryptic soy agar (TSA) plates. The plates were incubated in an inverted position at 60 °C for 48 h before the colonies on each plate were enumerated.

*C. sporogenes* spores were recovered using the same method where any food sticking on the surface each pouch was washed thrice in autoclaved distilled water to remove any residues followed by cutting on an edge using a sterile knife to empty the contents into a universal tube with peptone solution (0.1%, 9 mL) (Fort Richards, Auckland, New Zealand) followed by serially diluting and plating onto tryptic SBA plates for enumeration. The plates were incubated in an inverted position at 37 °C for 48 h in anaerobic chambers (Section 2.1) followed by enumeration.

### 2.8. Statistical Analysis

Three processing replicates were used for the *L-value measurements to determine the coldest spot for inoculation in subsequent trials. Once the cold spots were determined, three processing replicates were included for the inoculation studies using both strains of spores and three technical replicates were used for plating each sample. The significant differences among the *L* values and the spore numbers were analyzed using one-way ANOVA followed by post hoc analysis using Tukey’s test (Minitab, version 19). Microsoft Excel was used to compute the average and standard deviation for graphical and tabular representation.

## 3. Results and Discussion

### 3.1. Determination of Cold Spots for Microbial Innoculation

The cold spots in this study were determined using the difference in browning as a result of Maillard reaction as an indicator of time-temperature exposure [21]. Maillard reaction involves a reducing sugar (e.g., ribose) that condenses with a compound possessing a free amino group (e.g., amino acid) to give a series of reactions and products, that impart the brown color as a result of one of the products called M2. Chemical marker M2 has been reported as an effective tool to monitor heating patterns of foods in microwave sterilization [21].

The mashed potato (food model) showed a visual difference in the extent of browning as expected. Lightness (*L) values were found to be the most appropriate method to identify the difference heat exposure acquired by the surface of mashed potato (food model) as also previously reported [21]. The identification of cold spots was done on the surface of the mashed potato (food model) on each tray which was divided into nine spots for the measurement of *L values [8]. The coldest spot on each tray was determined by analysing the results of three processing runs. Cold spots detected were different for the two types of processing regimes at R-65 (Table 3a) and R-121 (Table 3b). Processing using hot water at 121 °C showed uniform browning across the nine regions in each tray (*p* > 0.05) (Table 3b).

**Figure 3:**
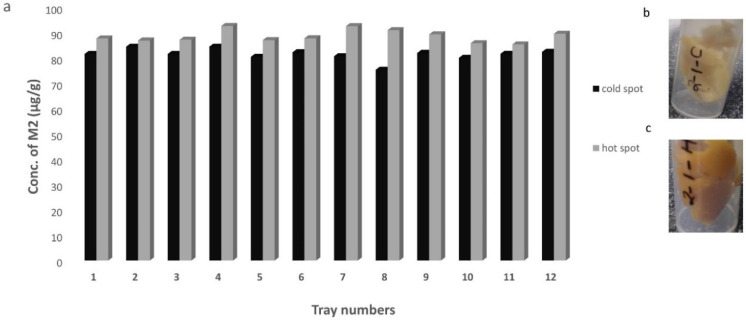
Concentration of M2 (μg/g of mashed potato) at the hot spot (grey bars) and the cold spots (black bars) determined on each tray (**a**) and visual representation of as an example of samples from the cold spot (**b**) and hot spot (**c**) scooped out from a mashed potato tray after CiMPAS processing at R-121. Similar letters among the bars indicate no significant difference (*p* > 0.05).

These results were in agreement with a previous study where the change in chemical marker (M2) as measured indirectly by *L values had shown temperature sensitivity that fits the Arrhenius relationship, which is a commonly used model to simulate the impact of temperature change on the reaction rate constants [21]. At the same time, Bornhorst et al. [21] also showed that the change in color or browning reached saturation after 100 °C due to a rapid rate of color formation. Similar uniform browning was observed with the current study with R-121 and hence, the cold spots were determined using a regime R-65 where the temperature of hot water was 65 °C. There was no significant difference across the nine spots measured on the control tray. However, the difference in *L values (9 spots/tray) post-processing in all the other trays (1–12) was due to Maillard reaction end products whose formation and concentration is affected by thermal exposure. Though the *L values among the 9 spots after R-121 on each tray were not significantly different (*p* > 0.05) from each other, the regions showing the highest and lowest *L value on each tray were further sampled (1 g) to be analysed using HPLC for the key Maillard intermediate product M2. For example, for tray 1, spot 1 was the hot spot as it showed an *L value of 55.1 ± 9.9 whereas the spot 3 was taken as the cold spot as it showed an *L value of 50.4 ± 1.7. Similarly, from each tray the spot showing highest *L value was chosen as the cold spot and the spot showing lowest *L value was taken as a hot spot. After HPLC analysis, the concentration of M2 was found to be significantly higher (*p* < 0.05) at the hot spot in each tray as compared to the cold spot (Figure 3b).

The accumulation of M2 was on the higher side on each hot spot as compared to the cold spot and hence it was concluded that verified that the change in browning (though not significant, *p* > 0.05) is a result of M2 formation and the lighter regions are still indicative of a cold spot for inoculation to check the inactivation locally. In the current study, the *L*-values were analysed on the surface of the tray and there is a possibility that the cold spots could be in the interior regions of the tray.

### 3.2. Thermal Resistance of C. sporogenes and G. stearothermophilus Spores in Mashed Potato and Milli-Q Water

**Figure 4:**
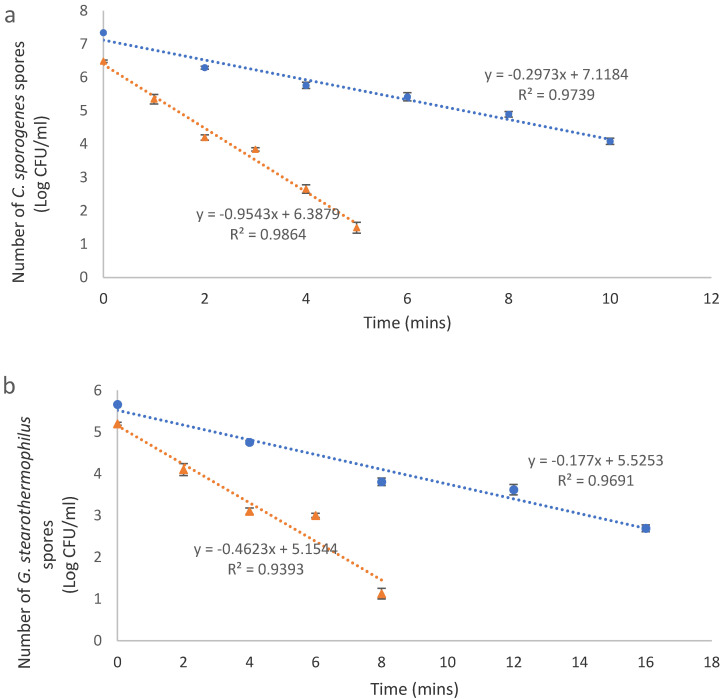
D121 °C values of *C. sporogenes* (**a**) and *G. stearothermophilus* (**b**) spores in autoclaved Milli Q water (triangles) and mashed potato food model (dots).

Decimal reduction time or D value is the exposure time required to achieve the killing of 90% or 1 Log CFU/mL of the living population of microbes at a predefined and controlled temperature [22]. Graphically, the D value is the inverse of the slope of the curve fitting the plot of the log10 value of the number of living cells against time. The D121 °C values of *C. sporogenes* spores were found to be 3.4 and 1.0 min in mashed potato and 0.1% peptone water, respectively (Figure 4a). On the other hand, the D121 °C values were found to be 5.6 and 2.2 min in mashed potato and 0.1% peptone water, respectively (Figure 4b).

The thermal resistance as evaluated at 121 °C for *C. sporogenes*, as well as *G. stearothermophilus* spores, varied significantly when the medium of inoculation was different (*p* < 0.05). This agrees with previous work by researchers, which indicates that the resistance of bacterial spores can be different attributing to several conditions including the food composition [23,24,25]. The current results agree with these previous indicates the importance of conducting a challenge test in food systems for validation.

### 3.3. Effect of CiMPAS on Inactivation of Spores

#### 3.3.1. Inactivation of *G. stearothermophilus* Spores

**Figure 5:**
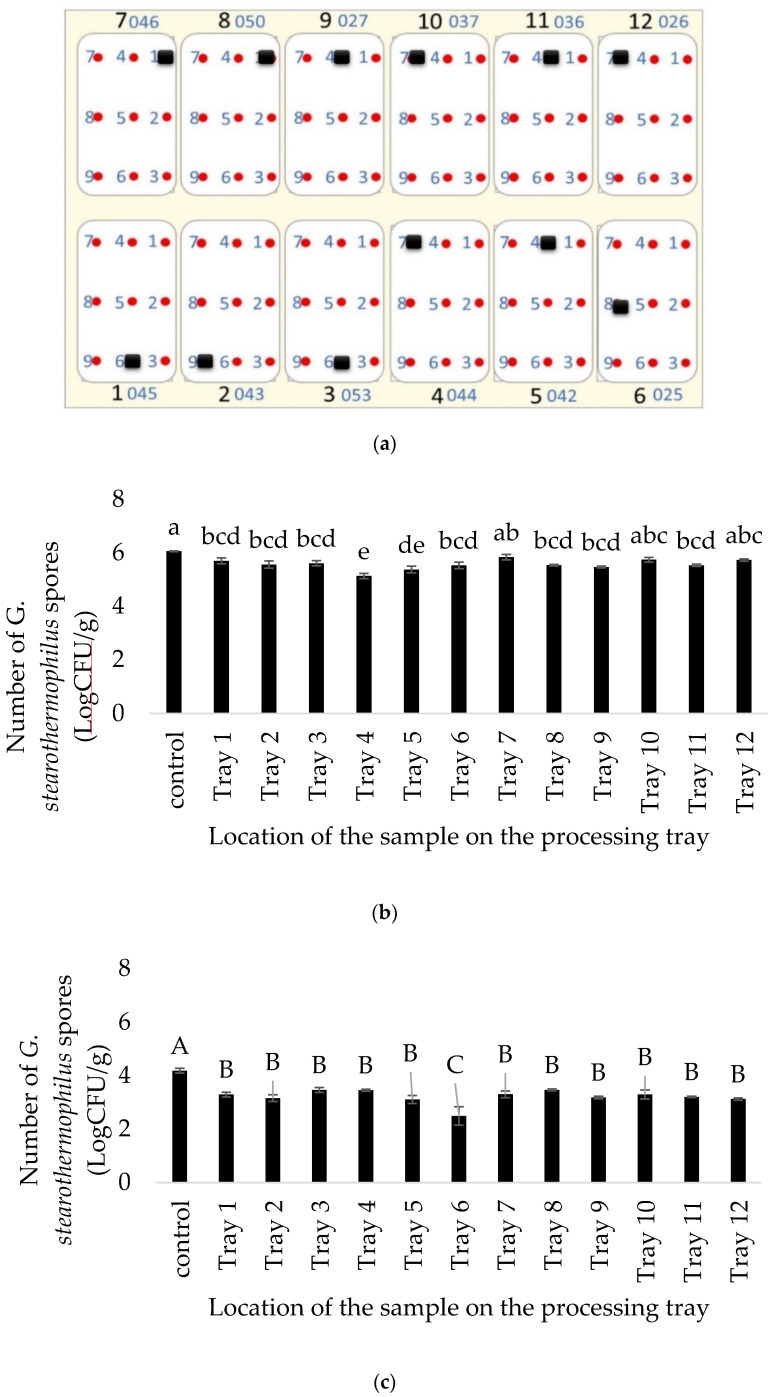
Inoculation map for spore pouches (*G. stearothermophilus*), where the set up represents a carrier tray with 12 packaging trays and the black squares shows the cold spot for pouch inoculation (**a**) *G. stearothermophilus* spores surviving post CiMPAS processing (R-121) using pouch inoculation (**b**) and whole tray inoculation (**c**) Different letters in each graph indicate a significant difference (*p* < 0.05); na = Not applicable for control.

*G. stearothermophilus* spores inoculated in pouches were placed in specific cold spots on each packaging tray as shown in Figure 5a for the spot inoculation (Figure 5b) and the inactivation was compared with the results from whole tray inoculation (Figure 5c).

All the trays showed inactivation within the detection limit of 2 Log CFU/g. The overall inactivation range for spores inside the spore pouches placed on the coldest spot on each tray (Figure 5a) ranged from 0.2 to 0.9 Log CFU/g (Figure 5b). On the other hand, the inactivation was equivalent to a range of 0.9 to 1.7 Log CFU/g when the whole trays with mashed potato (food model) were inoculated with *G. stearothermophilus* spores (Figure 5c). Tray 6 showed a significantly higher (1.7 Log CFU/g) inactivation of spores using the whole tray inoculation method as compared to all the other trays (Figure 5c). Using pouch inoculation, significant differences among the 12 trays could be detected (Figure 5b) whereas using whole tray inoculation, only tray 6 was different from the 11 other trays (Figure 5c). This further indicates that inoculation using pouches at specific locations might enable the detection of differences that the whole tray inoculation might not.

Inactivation of *G. stearothermophilus* by microwave sterilization has not previously been reported. One of the potential reasons would be the high thermal resistance of these spores, however, in the current study, they were an effective indicator of the difference in inactivation attributing to the thermal exposure.

#### 3.3.2. Inactivation of *C. sporogenes* Spores by CiMPAS

The pouches containing mashed potato inoculated with *C. sporogenes* spores were placed on three putative locations (including cold, hot and a spot that showed intermediate *L value) on each of the 12 processing trays. More than 7 Log reduction of *C. sporogenes* spores with a detection limit of 10 CFU/g was achieved post CiMPAS at R-121 on each of these three spots (Table 4). Since the D121 °C values (or the time required for 1 Log reduction) of *C. sporogenes* spores could vary from 0.9–1.4 min, which indicates that to achieve inactivation equivalent to 7 log CFU/g, these spots would have been exposed to an equivalent temperature of 121 °C for 6.3–9.8 min. Enrichment analysis (data not shown) indicated the presence of low numbers of survivors (1–10 CFU/g) in each tray.

**Figure 6:**
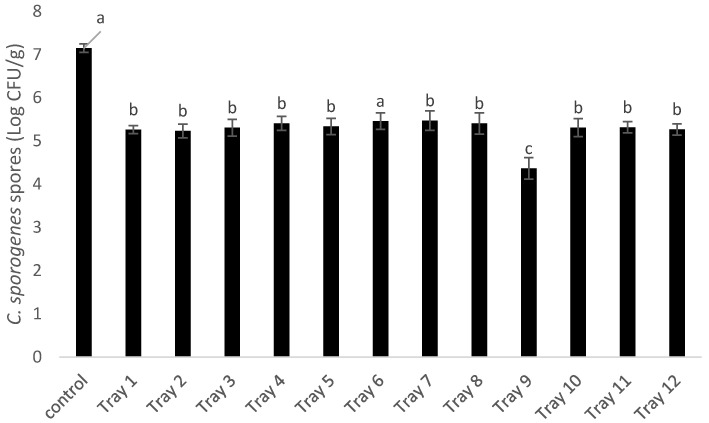
Loci for inoculation for spore pouches (*C. sporogenes*), where the set up represents a carrier tray with 12 packaging trays and the black squares shows the cold spot for pouch inoculation(a) *C. sporogenes* spores surviving post CiMPAS processing in pouches inoculated at the coldest spot on each tray after processing through R-65. Each bar represents the average e ± standard deviation (*n* = 9) including three technical and three processing replicates. Note: Similar letters among the bars indicate no significant difference (*p* > 0.05)

Inactivation of *C. sporogenes* spores has been previously reported using microwave sterilization (915-MHz, 10-kW pilot-scale MW system developed at Washington State University) when inoculated in pre-treated sliced beef (heated in boiling water with 0.5% salt) in gravy in 7-oz trays, where about 8 Log reduction was observed with F0 of 6 and 3 Log reduction with an F0 of 3 [26]. Since *C. sporogenes* are less resistant as compared to *G. stearothermophilus* spores, they showed a much better level of inactivation (>7 Log CFU/g), which is one of the requisites of commercial sterilization. Though the inactivation was uniform (Table 4), to further see the actual difference between numbers, a different CiMPAS regime with hot water at 65 °C but an increased number of passes to 12 and an increased microwave power of 22 kW was used for processing mashed potato while inoculated with *C. sporogenes* spore pouches in the coldest location as shown in Figure 5a. CiMPAS using R-65 showed significantly lower inactivation of *C. sporogenes* spores as compared to R-121 as expected (Figure 6).

**Figure 7:**
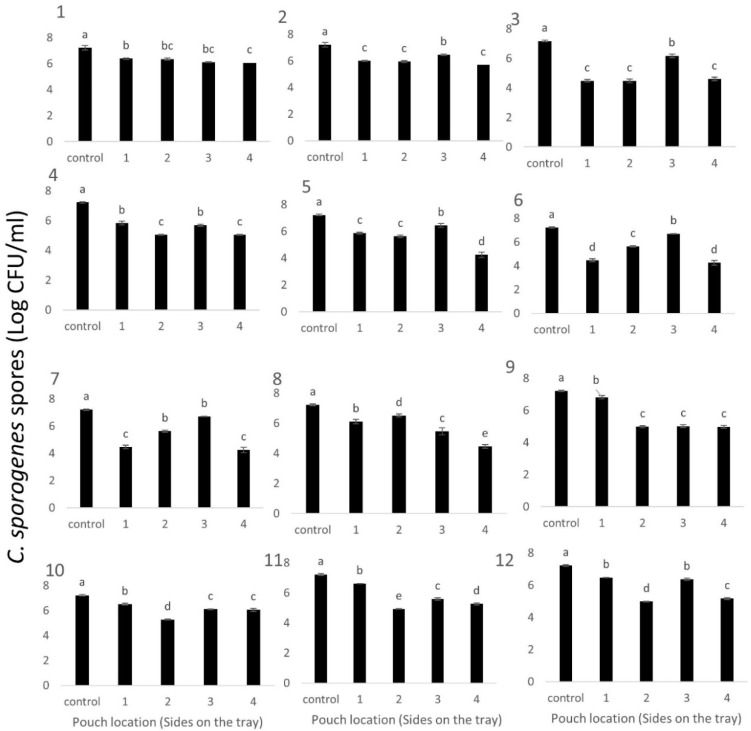
C. sporogenes spores in pouches surviving the CiMPAS processing (R-65) when placed vertically on four sides on each tray (**1**–**12**). Note: Similar letters among the bars indicate no significant difference (*p* > 0.05)

Tray 9 showed significantly higher inactivation in all three processing runs (Figure 6). The consistency of results in three different processing replicates indicated that the heat distribution was not variable enough to cause any difference in inactivation of *C. sporogenes* spores and pouches could be used as an effective method when the inactivation potential at a particular location inside the food tray needs to be evaluated. In a subsequent trial, to help understand thermal exposure at the walls/sides of each tray, a pouch was placed in a vertical position against each of the four sides and processed using the same regime (R-65) and the surviving spores were enumerated (Figure 7).

The inactivation of *C. sporogenes* spores at all four different sides of each tray in this study was an assessment of any difference in thermal exposure. The results showed that the spore pouches placed near the region coming in direct contact with water, for example, side 2 in tray 12, consistently showed better inactivation (Figure 7). In this trial, the spore pouches were able to indicate the difference in thermal exposure. Hence, the current study supports the possibility of using spore-inoculated pouches and recovery of spores/vegetative cells to test the inactivation potential at quite precise locations.

The thermal resistance of *G. stearothermophilus* spores and *C. sporogenes* spores could significantly vary in a wide range of food products. For example, D121 °C values of *G. stearothermophilus* can range from 0. 9 to 8.5 min (average of 2.4 min) as determined after assessing the effect of different minerals, sporulation conditions and 18 different spore strains [27]. On the other hand, *C. sporogenes* spores have been reported to show a *D*_121_ value of 0.92 min in phosphate buffer [28]. *D*_121_ values of *C. sporogenes* spores in food has been reported to range from 1.2–1.4 min in asparagus substrate acidified with gluconolactone (GDL) [29] and 1.28 min in liquid (unnamed) media (pH 7.0) [30]. Mashed potato (food model) is a semisolid food matrix, therefore was selected to understand the influence of the specific matrix on the inactivation using CiMPAS. In the current study, the *D*_121_ values of *C. sporogenes* and *G. stearothermophilus* spores showed a significant increase of 2.4 and 3.8 min when in mashed potato as compared to Millli Q water, hence also supports this fact that the resistance would change based on the matrix. Hence, each time a new model of food is tested, a validation study with microbial inactivation should be separately conducted and for that purpose, use of spore pouches instead or alongside of whole food inoculation would enable testing according to spatial mapping in food trays.

Inactivation equivalent to >7 Log reduction of *C. sporogenes* spores using spore pouches as observed in the current study indicates that a minimum of 6 min of average exposure at 121 °C was received by each tray. In the current study, up to 2 Log reduction of *G. stearothermophilus* spores using pouch inoculation also indicated that more than 6 min of average exposure would be attained by each tray. Both these findings were consistently reproducible using the pouches that were inoculated with spores in mashed potato (food model). The spores were inoculated in mashed potato just to ensure the coverage of any food-induced masking or protective effect as it has been reported for milk, meat and food with a high-fat content [31,32]. The findings indicate that placing these pouches inoculated with the desired kind of bacterial spores might help to understand if there would be any inactivation at colder regions inside the food tray in contrast to whole tray inoculation which cannot take the worst-case scenario of the coldest spot in any tray. Pouches also have potential to be formulated with different food matrix and different spore strains according to the requirements of the thermal regime being analysed (after initial standardization trials).

For this study, the lightness values on the topmost layer were taken to indicate broadly the heating experience of the food column below. However, the coldest spot could be somewhere in the mid-layer of the tray. In future studies, lower layers of the mashed potato (food model) tray will be used as a subject to measure cold spots.

## 4. Conclusions

Bacterial spore pouches were developed as a method to evaluate thermal exposure on specific locations inside food trays. Two strains of bacterial spores, with a significant difference in their thermal resistance (D121 °C) were used in this study to evaluative the inactivation using CiMPAS as a case study. CiMPAS regime in its research stage was deliberately chosen to give conditions generating variation in thermal exposure that could generate cold and hot spots. A CiMPAS regime at 121 °C (6 passes, 12 kW) at 915 MHz, although not yet optimised, showed >7 Log reduction in *C. sporogenes* whereas a similar treatment at 65 °C showed <2 Log reduction on the cold spots which were pre-determined using the difference in color as a result of Maillard browning, where higher lightness values indicate less heat exposure. Inactivation equivalent to 1–2 Log CFU/g of *G. stearothermophilus* was obtained using the regime at 121 °C indicating that the spores in the pouches were inactivated based on their thermal resistance and hence the pouch itself did not act as a restriction to mask any effect. Bacterial spore pouches with food matrix inoculated with spores could be used as an effective analytical tool to understand inactivation potential at specific location to understand spatial distribution effects. As microwave sterilization is an emerging technology, this method could be effectively used as part of the validation regime where non-uniform heating is an issue.

## Figures and Tables

**Table 1:** The processing steps for R121.

Step Number	Processing Step	Time (s)	The Temperature of the Vessel (°C)	Transport Speed (cm/min)	Number of Passes	Microwave Power (kW)
1	Pre-Pressurise	~70	na	na	na	na
2	Preheat water in	~70	30	na	na	na
3	Preheat hold	1800	30	na	na	na
4	Preheat water out	~70	30	na	na	na
5	Hot water in and	~70	121	na	na	na
	Microwave on	10	121	na	na	12
6	Microwave + carrier drive	250	121	130	6	12
7	Hold time	330	121	na	na	na
8	Hot water out	~70		na	na	na
9	Cooling water in	~70	30	na	na	na
10	Cooling water hold	900	30	na	na	na
11	Cooling water out	~70	na	na	na	na
12	Venting	~70	na	na	na	na

Note: The temperature of the hot and warm water vessel was set at 121 and 30 °C, respectively. na = not applicable.

**Table 2:** The processing steps for R-65.

Step Number	Name of the Processing Step	Time (s)	Temperature of the Vessel (°C)	Transport Speed (cm/min)	Number of Passes	Microwave Power (kW)
1	Pre-Pressurize	~70	na	na	na	na
2	Preheat water in	~70	30	na	na	na
3	Preheat hold	1800	30	na	na	na
4	Preheat water out	~70		na	na	na
5	Hot water in	~70	65	na	na	na
	Microwave on	10	65	na	na	22
6	Microwave +carrier drive	500	65	130	12	22
7	Hold time	330	65	na	na	na
8	Hot water out	~70		na	na	na
9	Cooling water in	~70	30	na	na	na
10	Cooling water holding	900	30	na	na	na
11	Cooling water out	~70	na	na	na	na
12	Venting	~70	na	na	na	na

Note: The temperature of the hot and warm water vessel was set at 65 and 30 °C, respectively; na = not applicable.

**Table 3:** a: Lightness (*L) values on 9 spots of each processing tray when processed using R-65; b: Lightness (*L) values on 9 spots of each processing tray when processed using R-121.

Tray No	*L Values								
	Spot 1	Spot 2	Spot 3	Spot 4	Spot 5	Spot 6	Spot 7	Spot 8	Spot 9
a									
1	57.6 ± 0.3 ^b^	54.8 ± 0.2 ^ab^	60.9 ± 2.1 ^ab^	59.5 ± 1.0 ^ab^	60.3 ± 1.6 ^ab^	62.0 ± 1.2 ^a^	58.9 ± 2.9 ^ab^	60.1 ± 3.1 ^ab^	60.9 ± 3.1 ^ab^
2	61.0 ± 6.4 ^a^	60.54 ± 4.7 ^a^	62.4 ± 3.6 ^a^	62.3 ± 3.2 ^a^	62 ± 3.5 ^a^	62.8 ± 2.0 ^a^	61.8 ± 1.2 ^a^	62.7 ± 0.7 ^a^	63.5 ± 1.7 ^a^
3	59.7 ± 1.8 ^abc^	56.1 ± 3.9 ^d^	61.3 ± 1.0 ^ab^	61.1 ± 1.3 ^abc^	58.1 ± 3.5 ^cd^	62.8 ± 1.0 ^a^	60.5 ± 1.4 ^abc^	59.3 ± 1.1 ^bc^	62.8 ± 0.7 ^a^
4	61.8 ± 3.4 ^ab^	58.06 ± 3.7 ^b^	61.1 ± 4.8 ^ab^	61.08 ± 4.8 ^ab^	61.2 ± 1.3 ^ab^	59.72 ± 0.8 ^ab^	63.2 ± 0.8 ^a^	61.5 ± 2.5 ^ab^	61.6 ± 2.5 ^ab^
5	61.3 ± 3.3 ^a^	58.8 ± 1.9 ^a^	60.1 ± 3.5 ^a^	62.4 ± 2.7 ^a^	61.7 ± 2.7 ^a^	61.8 ± 2.6 ^a^	60.5 ± 2.4 ^a^	60.4 ± 3.2 ^a^	61.2 ± 2.4 ^a^
6	58.7 ± 3.6 ^a^	56.5 ± 4.2 ^a^	57.4 ± 3.5 ^a^	60.0 ± 3.2 ^a^	57.3 ± 3.3 ^a^	58.0 ± 2.1 ^a^	58.9 ± 4.1 ^a^	60.6 ± 3.0 ^a^	57.8 ± 3.0 ^a^
7	65.5 ± 2.2 ^a^	62.9 ± 0.6 ^bcd^	65.2 ± 0.9 ^a^	65.4 ± 0.8 ^a^	62.9 ± 1.2 ^bcd^	63.8 ± 0.4 ^abc^	64.0 ± 1.8 ^ab^	61.8 ± 1.3 ^cd^	61.4 ± 1.8 ^d^
8	65.8 ± 1.5 ^a^	62.0 ± 1.6 ^bc^	63.0 ± 2.4 ^abc^	64.9 ± 2.7 ^ab^	61.0 ± 0.5 ^c^	62.3 ± 3.2 ^bc^	63.4 ± 3.7 ^abc^	61.2 ± 1.1 ^bc^	62.2 ± 2.7 ^bc^
9	62.6 ± 1.7 ^a^	62.5 ± 3.5 ^a^	62.1 ± 3.6 ^a^	64.0 ± 1.6 ^a^	62.4 ± 3.1 ^a^	63.4 ± 3.5 ^a^	63.7 ± 1.3 ^a^	63.4 ± 1.8	63.6 ± 2.9 ^a^
10	63.3 ± 1.5 ^a^	61.9 ± 3.0 ^a^	62.1 ± 4.3 ^a^	63.6 ± 1.4 ^a^	61.8 ± 3.2 ^a^	61.5 ± 4.7 ^a^	63.9 ± 2.5 ^a^	62.0 ± 2.9 ^a^	62.5 ± 4.0 ^a^
11	63.5 ± 0.8 ^ab^	61.4 ± 2.7 ^bc^	60.3 ± 4.5 ^a^	64.7 ± 0.8 ^a^	62.3 ± 2.0 ^a^	60.6 ± 2.8 ^a^	63.1 ± 0.4 ^a^	63.0 ± 1.4 ^a^	61.5 ± 2.4 ^a^
12	65.7 ± 1.4 ^ab^	61.7 ± 2.8 ^bc^	62.6 ± 3.9 ^abc^	66.2 ± 1.2 ^a^	61.0 ± 3.5 ^c^	63.8 ± 2.7 ^abc^	66.4 ± 1.1 ^a^	62.7 ± 2.7 ^abc^	63.9 ± 3.0 ^abc^
**b**									
1	55.1 ± 9.9 ^a^	51.7 ± 5.8 ^a^	50.4 ± 1.7 ^a^	56.0 ± 9.3 ^a^	54.1 ± 7.0 ^a^	52.3 ± 1.9 ^a^	51.0 ± 6.8 ^a^	51.2 ± 6.7 ^a^	50.2 ± 4.5 ^a^
2	44.3 ± 1.4 ^a^	45.3 ± 2.9 ^a^	46.5 ± 3.8 ^a^	45.7 ± 0.5 ^a^	48.6 ± 1.6 ^a^	47.7 ± 2.5 ^a^	43.7 ± 1.2 ^a^	44.6 ± 1.2 ^a^	46.9 ± 2.7 ^a^
3	50.1 ± 6.2 ^a^	52.2 ± 3.6 ^a^	53.1 ± 9.5 ^a^	51.3 ± 5.3 ^a^	54.5 ± 5.8 ^a^	55.5 ± 7.4 ^a^	49.3 ± 5.8 ^a^	52.7 ± 6.9 ^a^	53.8 ± 7.1 ^a^
4	55.5 ± 5.6 ^a^	51.0 ± 7.6 ^a^	54.2 ± 3.7 ^a^	55.8 ± 8.4 ^a^	52.9 ± 4.6 ^a^	53.0 ± 6.7 ^a^	52.0 ± 8.6 ^a^	50.9 ± 6.7 ^a^	49.35 ± 4.9
5	54.8 ± 6.6 ^a^	51.8 ± 5.6 ^a^	48.3 ± 6.6 ^a^	56.7 ± 9.3 ^a^	55.8 ± 6.8 ^a^	56.2 ± 7.5 ^a^	52.8 ± 9.6 ^a^	53.3 ± 6.1 ^a^	52.1 ± 8.3 ^a^
6	51.2 ± 4.0 ^a^	51.1 ± 9.0 ^a^	53.2 ± 9.4 ^a^	50.8 ± 2.5 ^a^	54.2 ± 4.3	55 ± 1.5 ^a^	46.7 ± 5.3 ^a^	50.5 ± 6.2 ^a^	51.5 ± 5.1 ^a^
7	55.4 ± 7.4 ^a^	51.4 ± 4.3 ^a^	52.9 ± 7.3 ^a^	56.0 ± 6.1 ^a^	53.9 ± 4.2 ^a^	53.1 ± 7.4 ^a^	54.1 ± 5.7 ^a^	50.2 ± 4.7 ^a^	53.3 ± 6.7 ^a^
8	52.1 ± 5.3 ^a^	53.4 ± 4.0 ^a^	52.4 ± 6.2 ^a^	53.8 ± 6.6 ^a^	54.5 ± 6.6 ^a^	55.4 ± 8.6 ^a^	51.0 ± 7.5 ^a^	53.3 ± 7.5 ^a^	54.2 ± 9.2 ^a^
9	57.2 ± 8.5 ^a^	53.3 ± 5.6 ^a^	50.8 ± 3.7 ^a^	55.3 ± 9.4 ^a^	54.5 ± 4.4 ^a^	52.7 ± 5.0 ^a^	54.9 ± 6.4 ^a^	51.0 ± 5.5 ^a^	50.2 ± 6.0 ^a^
10	54.4 ± 5.8 ^a^	52.9 ± 4.6 ^a^	48.5 ± 5.7 ^a^	57.2 ± 5.6 ^a^	53.6 ± 4.0 ^a^	50.8 ± 4.9 ^a^	54.4 ± 3.8 ^a^	49.1 ± 3.0 ^a^	50.0 ± 4.6 ^a^
11	53.4 ± 3.5 ^a^	53.1 ± 5.8 ^a^	55.6 ± 7.2 ^a^	51.2 ± 3.9 ^a^	53.9 ± 3.5 ^a^	51.0 ± 3.2 ^a^	50.0 ± 1.7 ^a^	52.0 ± 4.6 ^a^	48.8 ± 1.8 ^a^
12	48.7 ± 3.2 ^a^	52.6 ± 4.1 ^a^	53.0 ± 4.6 ^a^	51.9 ± 4.0 ^a^	55.4 ± 5.2 ^a^	55.2 ± 3.8 ^a^	50.9 ± 5.4 ^a^	50.0 ± 3.9 ^a^	51.8 ± 4.7 ^a^

Note: The same letters in superscript in the rows represent no significant difference (*p* > 0.05).

**Table 4:** Inactivation of *C. sporogenes* spores at three different locations on each tray after Coaxially induced microwave pasteurization and sterilization (CiMPAS) (R-121).

Tray Number	Three Locations of Inoculation from TABLE 3a	Number of *C. sporogenes* Spores (Log CFU/g)		
		First Location	Second Location	Third Location
Control	1, 5, 9	7.2 ± 0.1	7.3 ± 0.1	7.5 ± 0.1
Tray 1	1, 3, 6	nd	nd	nd
Tray 2	1, 2, 8	nd	nd	nd
Tray 3	3, 5, 8	nd	nd	nd
Tray 4	4, 6, 9	nd	nd	nd
Tray 5	2, 4, 9	nd	nd	nd
Tray 6	2, 5, 9	nd	nd	nd
Tray 7	3, 5, 6	nd	nd	nd
Tray 8	4, 6, 9	nd	nd	nd
Tray 9	4, 6, 9	nd	nd	nd
Tray 10	2, 5, 9	nd	nd	nd
Tray 11	2, 5, 9	nd	nd	nd
Tray 12	2, 3, 5	nd	nd	nd

Note: nd = not detectable with the detection limit of 10 CFU/g.
